# Construction of the REACHES climate database based on historical documents of China

**DOI:** 10.1038/sdata.2018.288

**Published:** 2018-12-18

**Authors:** Pao K Wang, Kuan-Hui Elaine Lin, Yi-Chun Liao, Hsiung-Ming Liao, Yu-Shiuan Lin, Ching-Tzu Hsu, Shih-Ming Hsu, Chih-Wei Wan, Shih-Yu Lee, I-Chun Fan, Pei-Hua Tan, Te-Tien Ting

**Affiliations:** 1 Research Center for Environmental Changes, Academia Sinica, 128 Academia Road, Section 2, Nankang, Taipei 11529, Taiwan; 2 Department of Atmospheric Sciences, National Taiwan University No. 1, Section 4,, Roosevelt Rd., Taipei 10617, Taiwan; 3 Center for Humanities and Social Sciences, Academia Sinica, 128 Academia Road, Section 2, Nankang, Taipei 11529, Taiwan; 4 Department of Applied History, National Chiayi University, 85 Wunlong Village, Minsyong, Chiayi 62103, Taiwan; 5 School of Big Data Management, Soochow University, 70 Linhsi Road, Shihlin, Taipei City 111, Taiwan

**Keywords:** Palaeoclimate, Databases

## Abstract

This paper describes the methodology of an ongoing project of constructing an East Asian climate database REACHES based on Chinese historical documents. The record source is *Compendium of Meteorological Records of China in the Last 3000 Years* which collects meteorology and climate related records from mainly official and local chronicles along with a small number of other documents. We report the digitization of the records covering the period 1644–1795. An example of the original records is translated to illustrate the typical contents which contain time, location and type of events. Chinese historical times and location names are converted into Gregorian calendar and latitudes and longitudes. A hierarchical database system is developed that consists of the hierarchies of domains, main categories, subcategories, and further details. Historical events are then digitized and categorized into such a system. Code systems are developed at all levels such that the original descriptive entries are converted into digitized records suitable for treatment by computers. Statistics and characteristics of the digitized records in the database are described.

## Background & Summary

It is now well accepted that the climate of earth has been changing all the time, sometimes drastically and sometimes mildly, and could occur in rapid or slow pace throughout the earth history (e.g., Lamb, 1977)^1^. The earth climate is a highly complex, nonlinear system whose complete comprehension is still beyond the reach for climate scientists. Such a system is capable of becoming chaotic and unpredictable as exemplified by the aperiodic solution discussed by Lorentz (1967) who used the convective system equations developed by Saltzman (1962) to demonstrate brilliantly this nonlinear nature^2,3^. This means that the climate system can become chaotic and change drastically as time evolves with a given initial condition even without any external forcing^4–6^. But external forcings such as solar irradiance, volcanic eruptions, variations in Earth’s orbital parameters, and perturbations of gases and aerosols can certainly alter the energy balance of the earth system; and thus cause climatic change^7^.

Many scientific investigations have been carried out to understand the climate change, especially to sort out the natural and man-made variabilities of climate. One of the common methods is to perform climate model studies. Various climate models have been constructed and many of them are physics-based^8,9^. But in these models many processes have to be simplified due to either our ignorance or technical practicality^10^. One widely recognized problem is the cloud and aerosol process, which contributes the largest uncertainty in climate predictions using climate models^7,11^.

With such simplifications, how do we know that climate models produce reasonable (though understandably never exact) predictions? One way is to use these models to reproduce climates in the past that we know well, and compare the predictions and records. This raises another question: do we have good climate data for such a comparison? The answer depends on the time scale and resolution of the climate phenomenon in question, but since the focal point of this paper is the historical time, a time scale of a few thousand years, the answer is sadly no so far. In such a time scale, the signals of natural variation and that due to external forcing are not distinct and we need climate data of relatively high spatial and temporal resolutions to resolve ambiguities. The most abundant data in existence for such a purpose are environmental data derived from proxy sources such as stratigraphic, pollen, tree rings, and trace chemicals (e.g., stalactite/stalagmite, δ^18^O, etc. ). Previously, most of the data sets are stored separately and with nonuniform formats. PAGES2K Consortium has made an important work assembling 692 temperature-sensitive proxy records into an unified dataset for the reconstruction of global temperature anomalies in the Common Era^12^.

While these proxy data have provided for many useful studies, they often don’t have adequate resolution and/or areal coverage. In addition, all environmental data need to be correctly interpreted and the method of interpretation can be a source of ambiguity^5,13–15^.

In this regard, direct human records, if available, will be less ambiguous than environmental data if we can trust the honesty of the recorders. By honesty, we mean that the weather is indeed cold when a record written by a human says that it is cold, and the weather is indeed wet when the record says so. This is to say that human records have less interpretation problem than environmental proxy records. Of course, human records have their problems as well which will be addressed when appropriate.

The historical records have been utilized in various studies previously, especially in historical climatology and paleoecology for retrieving important information to reconstruct past climate and infer changes of species distribution in the last thousands or hundreds years for Asia^16–19^, Europe^20–23^ and South America^24^. However, the majority of these records was scattered around the world and owned by individual investigators or institutions. Recently, efforts have been made to compile the documentary data into databases such as Euro-Climhist^25^, TAMBORA^26^, and TEMPEST^27^ mainly in central Europe and England. There appears to be no consistent endeavor in Asia to systematically compile all those records into a coherent database.

This paper is to report our recent effort to construct a climate database based on historical documents of China in the past 3000 + years. This data set will be called *REACHES* standing for Reconstructed East Asian Climate Historical Encoded Series. This is an ongoing work and so far we have completed the reconstruction of the series for early Qing Dynasty (1644-1795 CE).

In this paper, the *pinyin* system is adopted for phonetic translation of Chinese historical and geographical names for the easiness of cross-comparison with other papers. On the other hand, the Chinese character set used in this paper is the traditional form (i.e., not simplified) as it is the one that is closest to the original records with least ambiguity. In the following, we will first give a brief sketch of the nature of the historical documents from which climate information can be extracted. Then we will give some specific examples of such records, followed by a detailed description of the methods and data records on how we construct the digital series based on these sources. Finally, some basic statistics and characteristics of the current dataset will be presented.

## Methods

### Climate information in Chinese historical documents as data source

Ever since the beginning of its historical time at around 1600 BCE (the beginning of Shang (商) dynasty which succeeded the legendary Xia (夏) dynasty), agriculture had been the main economic activity of China, and climate was one of the biggest factors impacting agricultural yields. Consequently, Chinese governments had been paying attention to any abnormal or unusual phenomena related to climate such as extreme cold or warmth, excessive rain or prolonged drought, strong winds, heavy snow, large hails, or even unusual lightning and thunder events, etc., and these were recorded by professional specialists such as imperial historians (太史) or imperial astronomers (欽天監)^28–30^. There are even direct records of weather such as Clear and Rain Records (晴雨錄) which recorded daily rainfall in a qualitative way but at time resolution to the hour level^18,31,32^ or Depth of Rain and Snow (雨雪分寸) which reported the measured depth of rain or snow accumulations^33^. These records are naturally useful materials for our climate reconstruction purpose. There are also other records closely related to climate conditions such as the failure of crops causing famine or the big locust breakout that destroyed crops from which the climate conditions at the time may be inferred. They were also abundantly recorded and useful records for our purpose. There are other types of records that can serve similar purpose and examples will be given when appropriate.

**Figure 1:**
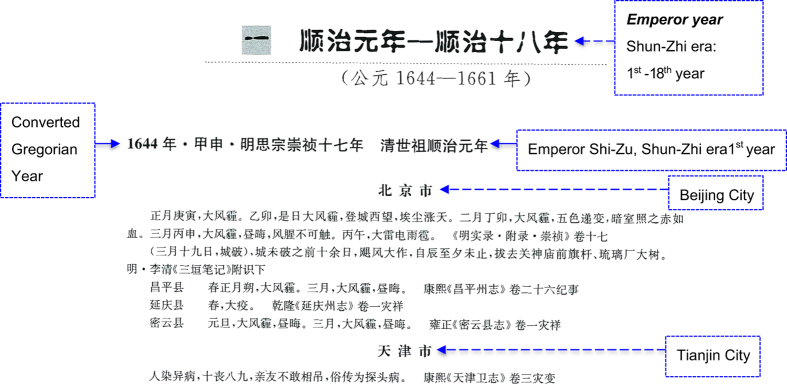
The image of the first record as published in *Compendium Vol. III 2nd Edition.*

Before Ming dynasty (1368–1644 CE), most of these climate-related records were documented in official chronicles compiled by government-appointed editorial boards consisting of officials and scholars. The chief editor was appointed by the emperor and was either a top-ranking official (and often the prime minister) or a widely recognized scholar official. With a few exceptions, the official chronicle of a dynasty was written by the above-mentioned editorial board of the succeeding dynasty after a dynasty had totally collapsed, so, for example, the Chronicle of Tang Dynasty was compiled and written by a Song dynasty (960-1279 CE) board after the Tang Empire was ended. The materials were based on the records compiled by imperial historians of the fallen dynasty and therefore usually of high uniformity and consistency. One example is the *shi lu* (實錄, records of facts) for which a sample will be shown later when discussing Fig. 1. Another is the *qi ju zhu* (起居注, records of imperial daily events). The style of such chronicles has been fairly fixed since the publication of Han Shu (漢書, The Book of Han) written by Ban Gu (班固, 32–92 CE).

A point of clarification must be noted here. While the above-said Han Shu whose style is normally recognized as the standard for all subsequent official chronicles to follow, it was not the first ‘official history’ written in China. There were other earlier chronicles or historical books before Han Shu. One well-known example is Shi Ji (史記) by Sima Qian (145-86 BCE) where similar records had been kept, but it was a personal endeavor of the historian himself instead of a government slated publication. There were also many histories of individual countries during the Zhou (周) Dynasty (circa 11^th^ century-771 BCE) and a well-known example is the Chun Qiu (春秋) by Confucius (551-479 BCE). Some of these earlier chronicles were written in more-or-less similar style like Han Shu (i.e., as annals) and some were not. Nonetheless, they are generally recognized as the “official histories”.

Official chronicles provide consistent records for climate series reconstruction, but their spatial coverage is fairly limited as the imperial historians tended to pay attention to events occurring in the national capitals and a few key locations deemed important to the country and ignore (or perhaps due to the lack of information) of places deemed insignificant. Also, the uniformity in style tended to result in generally terse records that left many details undocumented.

Beginning in Ming dynasty, the writing of local chronicles (Fang Zhi, 方志, or Difang Zhi, 地方志) became popular that greatly expanded the availability of documentary sources from which climate information may be retrieved. Depending on the size of their areal coverage, these records can be provincial, prefectural, city, county, or even a township. They often contain certain unusual weather or climate-related statements similar to those in the official chronicles and hence can serve as the purpose of climate information retrieval. To be sure, writing of local chronicles can be traced far back before Ming dynasty but it was not systematic. After Ming, such compilation became systematic and most sizable cities or counties as well as some prefectures had such records compiled. When collected together, these local records cover a large area with good spatial density. The entries often contain more lively descriptions than that in the official chronicles. Note, however, that the historians who compiled the local chronicles were usually not as professionally trained as those compiling official chronicles, and consequently the styles are less uniform and therefore care must be taken when deciphering their real meaning.

Both official and local chronicles have been published widely and many editions by different publishers exist, hence they are easily available for anyone interested in examining them. But the total volume of these documents is huge and to extract climate-related records from them is a truly challenging task. Fortunately, this task has been accomplished by a team of investigators in historical climate led by Zhang De’er and the result is the publication of *A Compendium of Chinese Meteorological Records in the Last 3,000 Years* (Zhang, ed., 1^st^ Edition 2004 and 2^nd^ Edition 2013 as a revised and expanded edition) (hereafter will be called *Compendium*)^34^. They had also added other records such as some private notes and stone tablets deemed appropriate in addition to those in the official histories and local chronicles. They examined 8,432 volumes of these documents and adopted 7,930 of them for compilation. *Compendium* begins with the records found in the inscriptions on the oracle bones of Shang Dynasty (1571-1046 BCE) and ends with those recorded in 1911 (the end of Qing Dynasty). While some records referred to events occurred in as early as 23^rd^ century BCE, most of them are derived from legendary descriptions of much later documents.

The *Compendium* is likely the most extensive compilation of written records of meteorological observations and climate-related events in the world, and thus invaluable in research of past climate variability. It is not only a relevant research material in China, but also widely used by Japanese scholars who still possess ability to literarily read and use Chinese characters^35^. However, the classic Chinese vocabularies have inevitably raised substantive difficulties in the readiness and interpretations of the records, especially for non-Chinese speakers. The inaccessibility does not only hinder the scientific community’s usage of the data published in the source, but also influence the transparency and reliability of the research based on the data^36^. *Compendium* contains the original entries of the historical documents in descriptive words in the style of Literary Chinese (*wen yan wen*, 文言文) which is a classical written Chinese language not used for daily conversation and one needs to be trained before one can understand such descriptions. Nearly all documents in China before the end of Qing Dynasty were written in such language. To render the meteorological records in *Compendium* usable with ease by researchers in the world, they need to be transformed into digital climate data series so that they can be easily treated by the present day computing technology. This is the motivation of the work to be presented in this paper. We have completed part of the digitization of the records but the work is ongoing. It is our plan to release the digitized series after the completion.

In the following sections, we will first describe a few detailed examples of the records to familiarize readers about their nature and contents, and followed by the methods we used to digitize them.

### An example of meteorological records in *Compendium*

*Compendium* consists of four volumes. Vol. I covers records from Shang (c. 1600BCE) to Yuan Dynasty (1279–1368 CE) and Vol. II covers Ming Dynasty (1368–1644 CE). Vol. III and IV contain records of Qing Dynasty (1644–1911 CE). Obviously, Ming and Qing, and especially Qing, have far more records than any previous periods. This appears to be a normal trend of historical records that there will be more of them the later the period is. This may indicate the increase of observations due to the advancement of technology (such as the spread of cellular phones may result in the increase of tornado reports in recent decades) or the increase of population so that more localities are established and hence more observational bases are available. Thus some caution must be taken when interpreting the climate series based purely on numerical statistics to avoid such data density bias.

Thus far we have completed the entering of the original records, deciphering the meanings, sorting of record types, determining the precise locations (latitudes and longitudes) and time, severity of the event (if appropriate), and derived several fundamental climate series out of them for Vol. III covering the period of 1644–1795. We will give more detailed descriptions of the records and methodology of digitization in the following.

Figure 1 shows a typical example in *Compendium* that contains the essential elements of such records. The portion containing Chinese characters is photocopied from *Compendium*, the English parts are our addition for explanation purpose. The original records use Chinese era year. For example, this page indicates the following records covering the period 1644–1661, corresponding to Chinese era period Shun-Zhi (順治) 1^st^ year to Shun-Zhi 18^th^ year. Shun-Zhi is the era name assigned by Emperor Shi Zu (清世祖) who was the first Qing emperor to govern the Chinese territory though not the founder of the dynasty. Some emperors had more than one era name during his reign. The first two lines contain the time information as explained by the blue box to the right of them.

The following line (the 3^rd^ line) indicates the specific year 1644 of which the following records belong. This is followed by the present name of location (Beijing City) where these events occurred as indicated by the blue box to the right. The following lines are all about what happened. The two lines followed can be translated as:

*“First month, the day Geng-Yin (see explanation of the day system in the next section), very windy and haze. The day Yi-Mao, this day was very windy and haze, wind-blown dust filled the skyline when we ascent to the city tower looking west. Second month, the day Ding-Mao, very windy and haze, (the haze) was changing in five colors (i.e., had many colors), and was as red as blood when it shone into a dark room. 3*^*rd*^ *month, the day Bing-Shen, very windy and haze, sky was dark during the day, wind had a foul smell (either smell fishy or bloody) that people dare not be in contact with. The day Bing-Wu, big thunder, lightning, and hail.* 《*Appendix of Ming Shi Lu in Chong-Zhen era*》 *Vol. 17”*

The information in the 《》brackets indicates the record source. 《*Ming Shi Lu*》(明實錄) is the contemporary records taken by Ming imperial historians. Chong-Zhen is the era name of the last emperor of Ming, Emperor Si Zong (明思宗), who committed suicide by hanging himself in 1644 in Beijing when the revolting army of farmers led by Li Zicheng (李自成) broke into the Imperial Palace. 《Ming Shi Lu》 contains records compiled by the central government, on which the descriptions in the official histories are based.

The line below this contains the following message:

“*(March 19, the city defense was broken), more than ten days before the city was broken through, there were stormy winds from the morning and continued to the evening. The wind fell the flag pole installed in front of the Guan Yu Temple’s gate and big trees in the Glass Factory” in footnotes (last part) of*《*San Yuan Bi Ji*》 *by Li Qing of Ming Dynasty.*”

Li Qing (1602–1683) was an official-scholar who lived through the exchange of the Ming and Qing dynasties and witnessed many important affairs of the time. 《San Yuan Bi Ji》(三垣筆記) contains his personal notes of these affairs he saw and heard when he was serving the government in Beijing. “Bi Ji” simply means notes. “San Yuan” means “three walls” but it is actually an astronomical term denoting the constellations around the Polaris. Li Qing used this term to represent Beijing area as Beijing was the national capital where the emperor dwelled and the Chinese traditionally considered the emperor as the Polaris in the human world.

The following 3 lines, on the other hand, are events noted in local records. They are translated in the following:

*“Changping County. Spring, 1*^*st*^ *month new moon day, very windy and haze. 3*^*rd*^ *month, very windy and haze, dark during the day. Kang-Xi era *《*Changping Zhou Chronicle*》 *Vol. 26 Event Records*

*Yanqing County. Spring, big plague. Qian-Long era *《*Yanqing Zhou Chronicle*》*Vol. 1 Disasters and Abnormalities.*

*Miyun County. New Year day, very windy and haze, dark during the day. 3*^*rd*^ *month, very windy and haze, dark during the day. Yong-Zheng era*《*Miyun County Chronicle*》* Vol.3 Disasters and Abnormalities”*

The localities mentioned in the above paragraph are now part of the Beijing City.

The next line indicates the location Tianjin City and the record below that belongs to Tianjin but shall not be translated here. Aside from the Shang Dynasty oracle bone inscriptions whose style and grammar are very different (and will not be discussed here), the vast majority of the records in *Compendium* are similar to this example.

### Digitization

**Figure 2:**
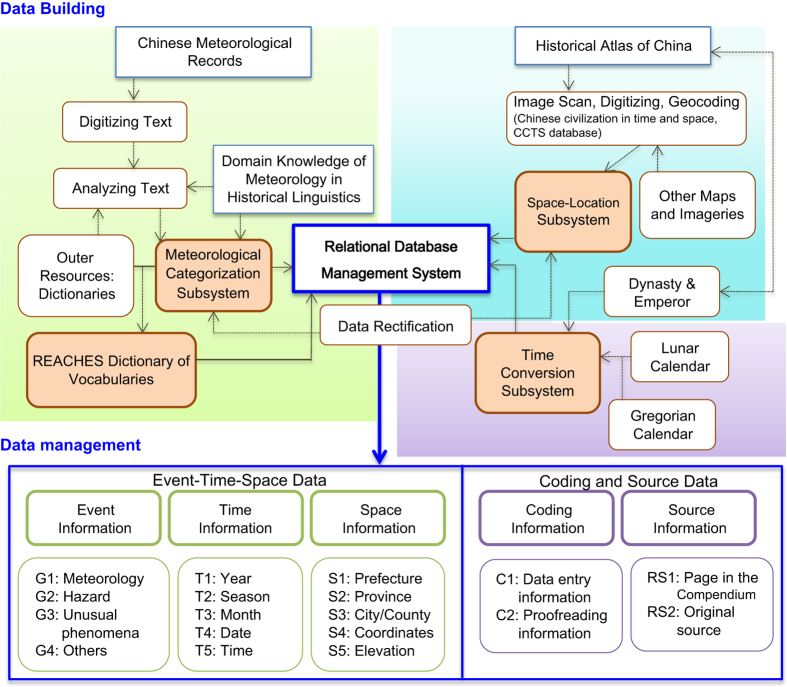
A flow chart of the *REACHES* database construction, showing the digitization process of the historical records in *Compendium* and how it interacts with the historical GIS and Lunar-Gregorian Calendar Conversion systems of Academia Sinica.

Our next step is to digitize the raw records as exemplified above. The general procedure goes like the following (Fig. 2). The records in *Compendium* are first electronically scanned (software Fujitsu ScanSnap SV600) and entered in computer through using optical character recognition software and then proofread by at least two research staff. For the digitization purpose, each record is given a record ID number containing initial six digits. For example, as illustrated in Fig. 1, the first lines quoted from《*Appendix of Ming Shi Lu in Chong-Zhen era*》 Vol. 17 was given a record ID as 1643-01, meaning that it is the first record on the page 1643. (Next quotation from 《*San Yuan Bi Ji*》 only appears in the second edition). Thus, the record under Changping county in the following line would be coded as 1643-02, and the one under Yanqing county would be 1643-03, etc. In addition, one record can sometimes contain multiple events occurring at different times of the year. For example, for the record in Changping county, the event occurred in the 1^st^ month of the year and the other happened in the 3^rd^ month should be separately treated in the system. We therefore distinguished them as 1643-02.000 for the 1^st^ month record, and 1643-02.001 for the 3^rd^ month record.

Then we break down each record into five basic elements, namely, the time, location (space), event, coder, and source which form the five dimensions of this record. We use a relational database management system (RDMS) framework to build a multi-tier categorization system as shown in Fig. 2. All details will be described in the Data Records Section.

### Code availability

The REACHES database only provides processed digits of the original records in *Compendium*, without their original texts. All rights of the original quoted records should belong to *Compendium* itself. The REACHES database is deposited at Data Citation 1 and Data Citation 2. The present database includes metadata, full sets of 49,714 L-2 records (will be explained below) during the early half of the Qing dynasty (1644–1795) of Vol. III, and three working sheets elaborating on the coding systems of the event categories. A complete version of the REACHES and documentation of the Chinese vocabularies especially the REACHES dictionary can be found in the REACHES homepage (http://reaches.rcec.sinica.edu.tw). It is open access but users need to apply for an account for activation. Right now the digitized version of Vol. III is from the first edition of *Compendium*. Differences between first and second editions for Vol. III and IV are very minor, less than two pages in total. Major differences appear in Vol. I and II. We will update all records to align with the second edition soon later. All new data will be uploaded constantly.

The Chinese speaking users who are interested in the original texts could easily trace the record ID in *Compendium* or trace back into their original historical sources. For non-Chinese speakers, all digitized data has been translated into English except the traditional Chinese vocabularies. They can easily retrieve the records of interests for analysis through setting multi-criteria (i.e., year, season, month, location and/or event category) for data retrieving.

## Data records

### Converting the time system

As described in the example record in the previous section, the time system used in all records are based on ancient Chinese system. The year is usually the Chinese era year. The month is usually the lunar calendar month (or its equivalent name). The day system is usually based on the Stems-and-Branches or *ganzhi* (干支) system which is a sexagenary cycle system^37,38^. For our digitization purpose, we convert the year, month and day to the western Gregorian calendar. The conversion is done by a Lunar-Gregorian calendar conversion system developed by Academia Sinica (http://sinocal.sinica.edu.tw, last update 20 July 2015).

Some of the records contain time information with resolution down to the hours. The hour system in the historical time was a 12-hour system called *shi chen* (時辰), for example, the first of these is *zi shi* (子時) which approximately corresponds to the time period 11 pm–1 am in the modern time system. Such time information in the records has been converted to the modern system.

### Locating the place

By “place”, we mean the location where a certain event occurred. We aim at finding the precise geographical location information, i.e., its longitude and latitude. The current names of the locations given in *Compendium* are based on the China’s 1987 revision on the administrative and jurisdictive figurations. The location names in the original records are, of course, that used at that specific historical period and need to be converted to modern names so that their longitudes and latitudes can be determined.

The location names in Chinese historical documents are sometimes quite confusing and hence it is necessary to exercise care in determining what and where they are. As is well known, a single location name can refer to many different places in different periods. For instant, the name Dongjing (東京), which simply means Eastern Capital, appeared many times in Chinese history during different dynasties. In Sui (隋) Dynasty (581–619 CE), the name Dongjing meant the city Luoyang (洛陽, the present city is located at 34°37′ N, 112°27′E, but the exact location changed from time to time in different dynasties although more or less in the same neighborhood). However, in Northern Song (北宋) Dynasty (960-1127 CE), Dongjing meant Kaifeng city (開封, presently at 34°48′N 114°18′E). Incidentally, the current capital of Japan, Tokyo, uses the same kanji characters 東京 (but pronounced as Tokyo) which also means Eastern Capital as the previous capital of Japan before 1869 was Kyoto which is located to the west of Tokyo.

The Jin (金) army invaded Kaifeng in 1127, thus formally ended the Northern Song Empire, and in 1153 changed the city’s name to Nanjing (南京, i.e., Southern Capital) as the capital of Jin Empire at the time was located in the north. During the early years of Ming Dynasty, Kaifeng was called Beijing (北京, Northern Capital) because the capital of Ming during the reign of its first emperor Tai Zu (明太祖) was at today’s Nanjing (32°03′N 118°46′E) which is located to the south.

Not only one place can have many different names in history, but there were also cases where several different places used the same name in the same period. This is hardly surprising because there were periods in Chinese history that several independent empires or kingdoms coexisted at the same time and some of them used same name for their own cities. This is exactly the same situation as the name London that is used by both the capital of the United Kingdom and a city in Ontario, Canada. Adding to the confusion is the common practice in ancient China to use the alias of certain locations that also need to be sorted out carefully.

To deal with such complicated historical geographic identification problems, a team of researchers in Academia Sinica developed a Chinese historical geographical information system (GIS) which allows us to determine the exact longitude and latitude of a location in a historical record with ease^39^. Upon the input of the time and the historical location name into this GIS, it will output the longitude and latitude of the location that are then entered in our database.

At least two rounds of calibration were conducted to assure the accurate temporal and spatial matching which was a very labor-intensive task to ensure data quality.

### Event categories

The most time-consuming task of all is the determination of the nature and degree of severity of the actual climate information contained in the record, and the categorization of them. The records in *Compendium* contain much diversified information, some are directly related to weather and climate (e.g., rain, temperature, wind) and some are events from which climate information can be inferred (e.g., flood, drought, famine, harvest, etc.). In many records, these events occurred together in a single line description (such as those described in Fig. 1) whereas for digitization purpose they should be stored in separate categories albeit they all belong to the same space and time coordinates. Furthermore, even among a kind of weather or climate phenomenon, there is a wide variety of types and severity. Take precipitation for example, not only there are different types of precipitation (rain, snow, hail, sleet, etc.) but each type of precipitation can have different varieties. For example, the category of “rain” alone, there can be heavy rain, medium rain, light rain, continuous rain, rain for a long period, etc. Similarly, the “snow” category can have heavy snow, blizzard, flurry, etc. To systematically tabulate these contents so that they can be subject to statistical analysis by computers, we developed a hierarchical data structure to categorize these events such that their relationship with the space-time and each other becomes clear.

The ontology of the categorization structure is based on the domain knowledge of meteorology and climatology in the context of historical linguistics. We first divide these events in four domains: (1) Meteorology, (2) Hazard, (3) Unusual Phenomena, and (4) Others. Table 1 gives a succinct description of these domains.

Examples of items in domains are also given in Table 1. These terms are self-explanatory and will not be deliberated here. Within each domain, there are Main categories. Within each main category there are Subcategories, for example, the main category Rain can have subcategories Rain, Snow, Hail, etc.

The domain “unusual” contains a wide variety of descriptions that are considered rarely happened but might have important impact. They are usually not directly related to weather and climate but could have some remote relations. For example, solar or lunar eclipse is in principle an astronomical phenomenon, but there are theories (e.g., Scafetta, 2012)^40^ that these celestial body arrangements could induce certain tidal motions in the atmosphere and oceans and hence could potentially impact the climate.

The lowest hierarchy of the digitized records is Vocabulary. This hierarchy contains the specific terms the authors of the records used to describe certain events. As indicated before, some records were written by professional government officials in charge of record keeping of weather, climate and other events while some were noted scholars who were not professional record keepers. The former group tended to write in more consistent manner and use consistent vocabularies in their descriptions. The latter group, being not necessarily professionally trained for record keeping, tended to use terms of their personal taste, therefore non-standard, in writing. For example, for describing rain event (as main category under precipitation domain), there were more than 40 vocabularies found in the Vol. III, e.g. 禱雨 (dao yu) for little rain, 斷雨 (duan yu) for light rain, 澍雨 (shu yu) for heavy rain, and 恆雨 (heng yu) for rain lasted for a long period, etc. These descriptions are obviously of importance and therefore it is necessary to further subdivide the subcategories into different types of rain through distinguishing the magnitude and duration of the rain events, such as light rain, heavy rain, continuous rain, or intermittent rain. Some descriptions used terms that were rarely seen in any other documents and are difficult to decipher. In such cases, professional historians were consulted.

Moreover, the meaning of the vocabularies also evolved throughout the dynasties. Not only different vocabularies can refer to same meaning, but one vocabulary can refer to many different things or phenomena. Thus careful interpretation is a prerequisite for the work; researchers often need to consult many other resources such as traditional Chinese dictionaries or historical literatures to pursue accurate interpretation.

To enhance transparency and facilitate reliability and to allow possible future modifications (for example, due to changes in interpretation), we have compiled a *REACHES Dictionary* which lists all vocabularies in the database such as those mentioned in the previous paragraph. In the dictionary, each vocabulary is explained and given a specific coder number that corresponds to the event codes so that users can track down how it is interpreted and defined. Presently the dictionary is in Chinese.

### Coder and source

In addition, the Coder (staff member who coded and proofread the records, dates, and completeness of the coding process, etc.) and Source dimensions (volume and page number, and original copied source etc.) were designed for data quality control and verification purposes. After all information was separately and systematically processed through the five dimensions, the related information under the same record ID number would be re-merged to constitute a full digit information chain for each record. Some of these terms are stored in Chinese at present.

The software tool for data entry and management is Microsoft Visual Studio 2013.

## Technical validation

The accuracy of the database that reproduces as faithfully as possible the information contained in the original descriptive records is of the utmost concern to this work. Several steps are taken to ensure this accuracy as described in the following.

### Source checking

We first check the accuracy of the original entries copied into *Compendium* from their original sources. At present, a comprehensive check is ruled out due to the large amount of records and the limitation of resources available to us. Instead, a limited sample check is performed. We chose to check the drought records in Vol. III of *Compendium*. In order to achieve standard confidence level, we randomly selected 808 records out of the total of 7,691to perform character-to-character check between that in *Compendium* and the original historical documents. However, a few sources listed in *Compendium* are not currently available in Taiwan and/or are not approachable through digital archives; we thus could only check 557 records (a sample size achieving 95% confidence interval) at this time. Not all inconsistencies are errors as some of them may be due to differences in editions. Some of the inconsistencies arise from the use of different character sets used in Mainland China (simplified) and Taiwan (traditional) and we do not count these as errors either. In the following, we only describe those inconsistencies that have potential impact on the interpretation of the climate contents in certain records. Some of the inconsistencies are associated with missing or different locations, thus would have impacted the geographical distribution of certain event while others are different dates which might impact the timing. In one case, a snow (雪) event is mistaken as a hail (雹) event possibly due to the similarity of the two characters. However, there are only 6 out of 9130 characters in these records that would have an impact on the climate contents, i.e., a rate of about 0.066%. If this rate can be applicable to all records in *Compendium*, then it indicates that the probability of inconsistency that would result in different interpretation of historical climate is likely to be very low.

Nevertheless, we will continue to check for the possible errors and make necessary corrections when resources become available in the future.

### Proofread and coding checks

After cleared the source checking and electronic processing, an entry becomes a record in our database and is subject to proofreading. A two-round proofreading process is enforced. Every record is proofread by at least one person, and sometimes by two or more. After the accuracy is confirmed, the record is coded according to the digitization scheme described above. The coding is first done by two different persons and the digitized data sets are compared with each other. If inconsistencies are found, a third or even fourth person would check the inconsistency until it is resolved. After that, the digitized record is accepted in the database.

### Data statistics

#### Site density

**Figure 3:**
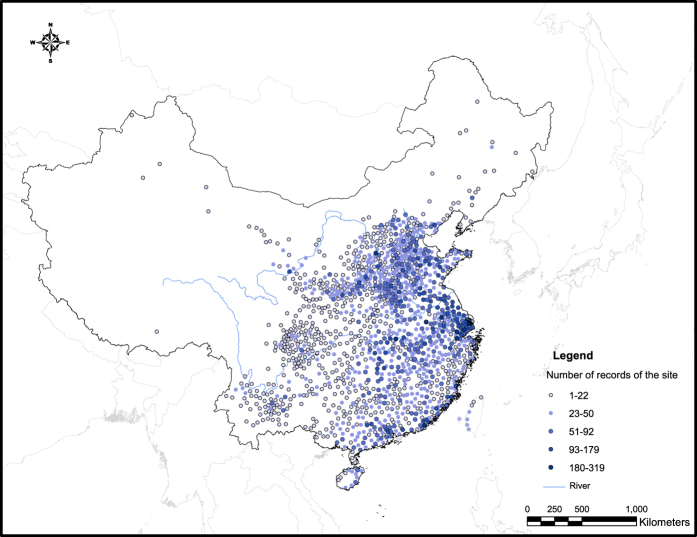
Map of geographical sites mentioned in the records in *Compendium* Vol. III. Colors represent the number of records of the site (n = 1,435).

There are in total 36,123 original records (Level-1, as L1-record) in the vol. III of *Compendium*, covering a total of 1,435 geographical sites in China. The spatial distribution of the sites that have records was extremely uneven, with high densities in the Yellow River and Yangtze River plains which were traditionally the political and economic centers of China (Fig. 3).

In contrast, the far northeastern, far west (Xinjiang), northwestern, and southwestern regions including Tibetan Plateau have rather sparse data coverage. Traditionally, these places had been frontier areas inhabited by minorities of China and it is understandable that few records were kept in the chronicles about them. The rest of regions have site density somewhere in between. It is clear that the sites that have records are mainly located in the eastern half of the current China.

Not only that the site density varies, the number of records at each site also vary widely, ranging from 1 (minimum) to 319 (maximum) L1-record(s). An uneven data distribution in both spatial and temporal domains is therefore present and any conclusions based on statistical analysis performed on these data sets should take this fact into account.

#### Time behavior of publication and record numbers

One of the common problems of using historical records is how to deal with the general trend of increasing number of publications as time evolves, assuming that more publication means more records. This is akin to a question often raised about the trend of tornado frequency in US: while there are indeed more reports about tornadoes in recent time than, say, 40 years ago, does this really indicate that there are more tornadoes recently than in the past, or it is merely because there are more reports due to, say, the convenience of mobile phones that makes reporting easier?

**Figure 4:**
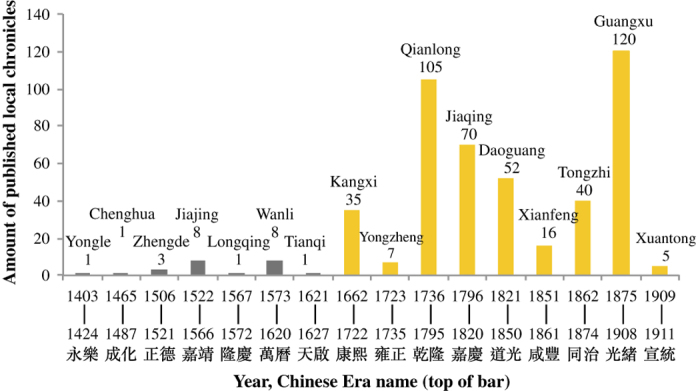
The number of local chronicles published as a function in the period 1403–1911. Gray bars indicate eras of Ming dynasty; Yellow bars indicate those of Qing dynasty. The Chinese characters at the bottom are Chinese era names.

Figure 4 shows the number of published local chronicles as a function of time^41^. It is apparent that the number of local chronicles published was much more in Qing Dynasty than in Ming. One therefore tends to suspect that this may cause data frequency bias which will favor the later period. However, it turned out that many of these later publications are simply the revised (and updated) versions of previous ones without really increasing significant amount of information contained in them. When we make a more refined look into the amount of records (representing the frequencies of significant events), we found the number is actually quite stable. We use the records of Sichuan Provincial Chronicles as an example to illustrate this point.

**Figure 5:**
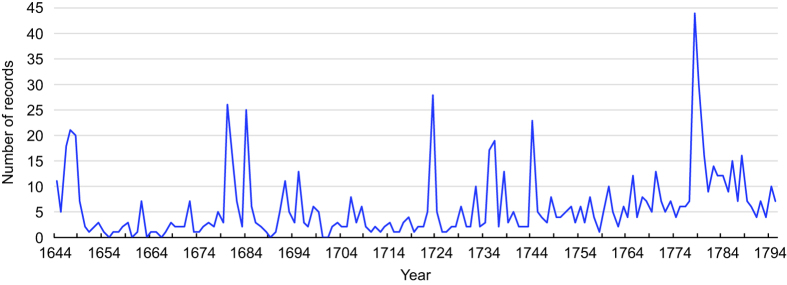
The number of L1-records in Sichuan Provincial Chronicles as a function of time in the period 1644–1795 (n = 906).

Figure 5 shows the number of records contained in *Sichuan Provincial Chronicle* as a function of time. While the number goes up and down throughout the period of 1644–1795 extending from early Qing to mid-Qing dynasty, it is seen that there is no obvious trend. This is even more remarkable considering that rebellion farmers led by Zhang Xianzhong (張獻忠) and then his followers during 1644–1659 controlled Sichuan and yet the number of records in that place was still substantial during such turmoil. Apparently, the recording of unusual events was not much deterred by the societal turmoil.

Another fact that may corroborate with the conclusion that the record number inflation with time is not such serious is that the number of L2-records (see explanation below) shows a decreasing, instead of increasing, long-term trend during this period. As we have seen in the example of Fig. 1, an original L1-record often contains more than one separate or discontinuous event, with same or different attributes, happening at different time of the same year. It is to the benefit of digitization to split these separate events into different records, which are called the Level-2, or L2-records. This results in a total of 49,714 L2-records in the database.

**Figure 6:**
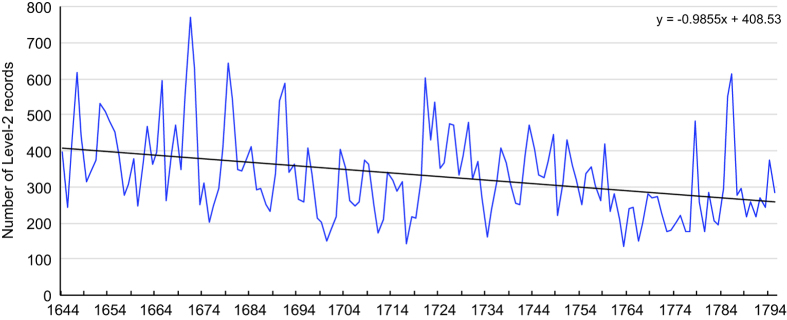
The number of L2-records as a function of time in the period 1644–1795 (n = 49,714).

For a single year, the number of L2-records varies from 134 (minimum) to 771 (maximum), obviously reflecting the yearly fluctuation of the events worth recording. Figure 6 shows the variation of the annual L2-record number as a function of time. We see that, unlike the increase in the publication number of local chronicles (Fig. 4), there is a general decline trend of the records. Thus, the feared inflation of records with time, if exists, must not be a serious issue in this database especially for the Qing dynasty.

This number stability probably stems from the fact that both official and local chronicles recorded only ‘significant’, ‘unusual’ or ‘remarkable’ events or things at the place. Events considered as normal, for instance, thunder and lightning in the summer or snow in winter in mid- to higher latitudes, were ignored. On the other hand, unusual or abnormal events, such as thunder and lightning in winter or snow in summer, were carefully noted and recorded. The small number nature of “unusualness” probably reduced the likelihood of record inflation. Our conclusion is: the fluctuation of the records in Fig. 6 most likely reflects the fact that the mid-1600s was a more active period with numerous significant or unusual events occurring than later years.

#### Record category population

**Figure 7:**
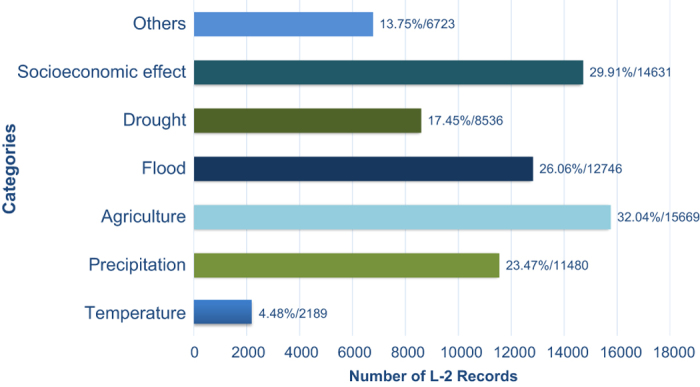
Number of L2-records in the six most populated categories. One record can contain more than one event (such as those described in Figure 1). Thus the percentage shows the actual categorical rate and therefore the total exceeds 100%.

Figure 7 shows the population distribution of record categories. It is seen that the greatest number of the records is concerned with the agricultural yield, i.e., crop growing conditions (32%), followed by socioeconomic effect (30%), flood (26%), precipitation (23.5%), drought (17.5%) and temperature (4.5%). This reveals that crop yield and adverse weather and climate condition that would threat the society and result in socioeconomic turmoil were of major concern of the country and thus of record writers.

**Figure 8:**
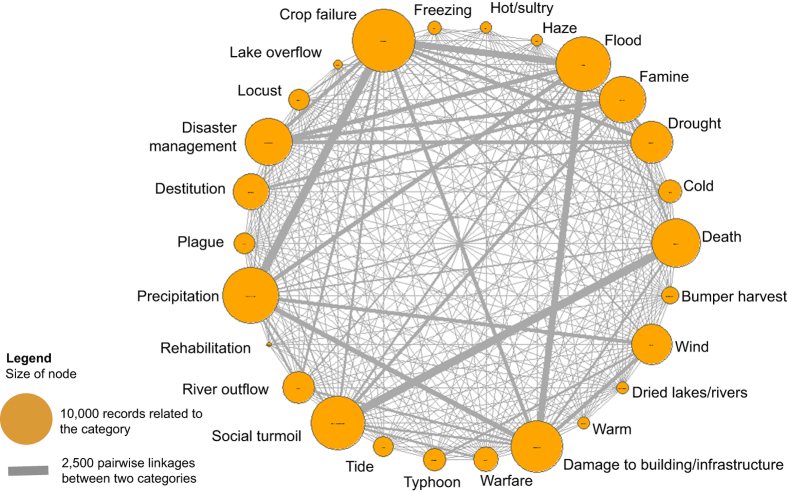
Network analysis chart of categories.

Relations between categories are further revealed by the network analysis chart^42^ (Fig. 8) using SAS 9.4 edge list statistics. The size of circle represents the number of records in a category. The same top five categories (bigger circles) as described in the previous paragraph are shown as they should be.

The thickness of the line connecting two different circles represents how frequent they are mentioned together in the records. This usually indicates that these categories are closely connected and may have some important associations. Thus, for example, crop failure is most frequently mentioned together with precipitation, flood, drought, famine, damage to building/infrastructure and cold climate; precipitation has close connection with crop failure, flood, damage to building/infrastructure, wind, and cold; social turmoil has the strongest connections with death, famine, flood, war/battle and crop failure.

The impact of disasters to the society is revealed by the fact that the disaster categories, e.g. flood, famine, and drought are all strongly connected with disaster management, i.e., actions that central or local governments took to mitigate the disaster consequences, such as distributing relief crops and goods in the most impacted areas, relief of tax, repair of destroyed dikes, and restoration of social order, etc. These connections reveal complicated relationships among all those records and the causalities. Upon further elucidation of these relations, it may be possible to use some records such as disaster relief measures as surrogates for climate conditions especially for locations where direct climate records are scanty.

## Usage notes

In the above, we have briefly introduced the broad contents of *Compendium* and described the process of digitizing the descriptive records into an electronic database so that modern computing technology can process them easily. This database can provide useful source materials for reconstructing past climate in East Asia, particularly that of the eastern half of China, for approximately the past 2000 years where Chinese historical chronicles were most systematic. It is understood, of course, that descriptive records cannot be as quantitative as instrumental data, however, they can provide convincing trends of climate change as they represent direct human observation of the environment and hence should suffer much less interpretation problem as often encountered when using environmental data.

As mentioned earlier, this is an ongoing work and we aim at the complete digitization of all records in *Compendium.* We will keep uploading new data at Data Citation 1 and Data Citation 2. We have also established several reconstructed climate series (temperature, precipitation, drought, locust, etc.) from this database and are analyzing them and will soon summarize our findings for publication.

## Additional information

**How to cite this article**: Wang, P. K. *et al*. Construction of the REACHES climate database based on historical documents of China. *Sci. Data*. 5:180288 doi: 10.1038/sdata.2018.288 (2018).

**Publisher’s note**: Springer Nature remains neutral with regard to jurisdictional claims in published maps and institutional affiliations.

## Supplementary Material



## Figures and Tables

**Table 1:** Hierarchical data structure of event categorization system in REACHES database.

Domain	Main Category	Subcategory (some examples)
Meteorology	Precipitation	rain, snow, hail, etc.
	Temperature	warm/sultry/freezing/cold
	Visibility	haze/fog/darkness
	Thunder, Lighting	thunder/lighting
	Optical	corona/rainbow/halo
	Wind	typhoon/tornado/wind
	Cloud	colored clouds
	Gas, Air	gas/colored gas
Hazard	Drought	drought/dry riverbank/dry well
	Flood	flood/tidal surge/ surge/tsunami/debris/
	Pest/Vermin	locust/other pests
	Crops	bumper/harvest failure, different species are distinguished.
	Disease	plague/malaria/herpes/dysentery
	Famine	famine/cannibalism
Unusual phenomena	Geophysical abnormities	earthquake/river course change/land cover change
	Precipitation abnormities: color	black rain/red rain/yellow rain
	Precipitation abnormities: plants	rained grains, nuts, flowers, etc.
	Precipitation abnormities: animal	rained fish, bugs, etc.
	Precipitation abnormities: metal	rained sulphur, black particles, etc.
	Acoustical abnormities	thunder without clouds
	Sun-related phenomena	solar eclipse, solar prominence, sunspot
	Moon-related phenomena	lunar eclipse, red moon
	Astronomical phenomena	new star (nova or supernova)
	Plant abnormities	abnormal growing conditions of plant, species are distinguished.
	Animal abnormities	abnormal animal conditions, species are distinguished.
	Socioeconomic turmoil	wars, unrest, human migration, disaster relief, human trafficking
Others	Unrecognized vocabularies	unclear ‘dragon’ description
